# Noninvasive Follicular Thyroid Neoplasm With Papillary-Like Nuclear Features (NIFTP): Achieving Better Agreement By Refining Diagnostic Criteria

**DOI:** 10.6061/clinics/2018/e576

**Published:** 2018-05-15

**Authors:** Venancio A.F. Alves, Kennichi Kakudo, Virginia LiVolsi, Ricardo V. Lloyd, Yuri E. Nikiforov, Vania Nosé, Mauro Papotti, Lester D.R. Thompson

**Affiliations:** I Departamento de Patologia, Faculdade de Medicina (FMUSP), Universidade de Sao Paulo & CICAP- Patologia, Hospital Alemao Oswaldo Cruz, Sao Paulo, SP, BR; II Department of Pathology and Laboratory Medicine, Nara Hospital, Kindai University Faculty of Medicine, Ikoma-city, Nara-ken, Japan; III Department of Pathology, University of Pennsylvania School of Medicine, USA; IV Department of Pathology, University of Wisconsin School of Medicine and Public Health, Madison, USA; V Department of Pathology, University of Pittsburgh Medical Center, Pittsburgh, Pennsylvania, USA; VI Departments of Pathology, Massachusetts General Hospital and Harvard Medical School, Boston, Massachusetts, USA; VII Department of Oncology, University of Torino, Italy; VIII Department of Pathology, Southern California Permanente Medical Group, Woodland Hills, CA, USA

Over the past decade, improvements in imaging technologies along with greater access to medical care have resulted in the discovery of neoplasms in a much earlier stage. This has contributed to a reduction in cancer mortality. However, an unintended consequence of early detection has been detection of lesions which present at an earlier pathologic stage of development, requiring modification of diagnostic criteria, as well as determining a more appropriate risk stratification to inform management.

The differential diagnosis between thyroid follicular epithelial neoplasms was classically based on architectural arrangement of either follicular or papillary growth. However, with the 2004 World Health Organization (WHO) Classification of Thyroid tumours 1, papillary carcinoma was defined by the presence of characteristic nuclear features, including nuclear crowding or overlapping, elongation, irregular contours, grooves, and chromatin clearing, and thus these features detected in a follicular-patterned neoplasm were diagnosed as a follicular variant of papillary thyroid carcinoma (FV-PTC). Interestingly, while capsular and/or lymphovascular invasion were the defining criteria for classifying a well-differentiated follicular neoplasm as follicular adenoma (no capsular nor lymphovascular invasion) or follicular carcinoma (presence of capsular and/or lymphovascular invasion), all epithelial neoplasms with the nuclear features of papillary carcinoma were diagnosed as papillary carcinoma, regardless of whether the neoplasms were encapsulated or invasive. Thus, distinctly different from follicular neoplasms, all encapsulated follicular variant of papillary thyroid carcinoma were classified as carcinomas (EFV-PTC), further subdivided based on the presence or the degree of capsular and/or lymphovascular invasion as *invasive* EFV-PTC or noninvasive EFV-PTC.

Noninvasive follicular thyroid neoplasm with papillary-like nuclear features (NIFTP) is the new terminology proposed for encapsulated follicular variant of papillary thyroid carcinoma (EFVPTC) without evidence of capsular and/or lymphovascular invasion.

Nikiforov et al. 2 reported the results of a histological review of digitalized histologic slides from 210 cases with thyroid nodules diagnosed as EFV-PTC by 24 experienced endocrine pathologists from seven countries. During initial evaluation, a very large group of histological variables was used by members of the panel with not neglectable discrepancies, even though all were expert thyroid gland pathologists. In a comprehensive and meticulous analysis, including 8 teleconferences and a face-to-face 2-day meeting, the members reached a consensus on the nuclear features of papillary thyroid carcinoma and defined the criteria for a noninvasive EFV-PTC, reclassified as noninvasive follicular thyroid neoplasm with papillary-like nuclear features (NIFTP): Full encapsulation or partial encapsulation with clear demarcation, follicular growth pattern (<1% papillae) and the nuclear features of papillary carcinoma, defined by a nuclear score of 2 or 3 (see visual guide) as the necessary positive findings. Further, exclusionary criteria were also developed: 1) the absence of lymphovascular and capsular invasion in an adequately sampled (i.e., completely submitted) tumor; 2) less than 30% of the tumor volume showing a trabecular, insular or solid architecture; 3) less than 3 mitoses/10 high power fields; 4) no necrosis identified; and 5) no psammoma bodies.

Employing these inclusion and exclusion criteria (see visual guide) resulted in a high interobserver agreement, yielding a sensitivity of 98.6% and specificity of 90.1% for non-invasive EFV-PTC patients (109 patients followed for 10-26 years) versus the invasive EFV-PTC patients (101 patients followed for 1-18 years). This separation was important for clinical purposes, as no patient from the noninvasive group developed any adverse event (no recurrence, no lymph node metastasis, no distant metastasis), whereas 12% of the patients from the invasive group developed some adverse event, including distant metastases in five patients, leading to death in two patients.

Molecular analysis for point mutations, gene fusions or rearrangements detected in the clear majority of papillary carcinomas were performed on 37 cases initially submitted for inclusion into group 1 (noninvasive EFV-PTC) and on 26 new cases of EFV-PTC incorporated as a validation set. Clonal molecular alterations were detected in 25 cases (68%), with *RAS* mutations the most common. None of the 5 cases excluded from the noninvasive group due to insufficient nuclear features had any of these mutations. In contrast, clonal mutations were detected in 21 (78%) of the lesions remaining in this group. Based on this study, Nikiforov et al., 2 proposed that the noninvasive EFV-PTC should be reclassified to noninvasive follicular thyroid neoplasm with papillary-like nuclear features (NIFTP).

The immediate impact of that publication was likely due to the multidisciplinary, international effort and systematic review of the group of neoplasms by a large group of pathology experts, which led to the relatively fast acceptance of the terminology applied to a class of thyroid neoplasms that may behave in a more indolent fashion than their fully malignant counterparts, influencing clinical management and reducing aggressive clinical treatments.

The 2017 WHO Classification of Endocrine Organ Tumours 3 included NIFTP in the group of the so-called “follicular-patterned neoplasms with borderline clinical behavior”, including NIFTP, along with follicular tumor of uncertain malignant potential (FT-UMP) and well differentiated tumor of uncertain malignant potential (WDT-UMP). While the latter two tumors include “questionable capsular or lymphovascular invasion,” NIFTP lacks any invasion in a tumor completely submitted for histological exam. The WHO classification also highlighted the strong association between the *RAS* and other *RAS*-like mutations and NIFTP, distinctly different from *BRAF* mutations frequently identified in other subtypes of PTC 3.

Within a year of publication, several series from different countries have been published, further corroborating and supporting the initial findings of a very low malignant potential tumor, without recurrence or metastasis.

Thompson 4 reviewed 94 cases of EFVPTC measuring 0.7 to 9.5 cm in diameter, with a mean tumor size of 3.3 cm, reviewing an average of 11.9 slides per cases. Capsular and/or lymphovascular invasion was found in 17 and no invasion in 77 cases. Lobectomy or thyroidectomy was the initial treatment combined with post-op radioablative iodine in 25 patients. Without any recurrence and no lymph node or distant metastases after a median follow-up of 11.8 years, Thompson concluded that encapsulated follicular variant of papillary thyroid carcinoma could be treated by conservative surgery alone and could be accurately reclassified as NIFTP based on the inclusion and exclusion criteria published.

Reviewing their series of 860 PTC >1.0 cm from Brazil, Rosario et al. 5 identified 129 cases which fulfilled the criteria for NIFTP, representing about 15% of all PTC cases. Based on original diagnoses, none of the patients reclassified as NIFTP had been given radioactive iodine therapy. No patient developed recurrence or metastasis during the median follow-up period of 72 months (range 12-146 months).

In an evaluation of large neoplasms exclusively (>4 cm), Xu et al. 6 showed that all 79 patients with NIFTP were without lymph node or distant metastases, including 25 patients managed without radioablation, followed for a median of 11.2 years. Furthermore, Rosario et al. 7 recently reported a favorable clinical course in 45 patients with NIFTP larger than 4 cm, where there was only 1 patient who developed lymph node metastasis, but this patient had a concurrent microscopic papillary carcinoma in addition to the NIFTP.

By contrast, not all recent reports show a completely benign clinical course, validating the classification of this lesion as a borderline or low grade tumor as proposed by Nikiforov et al. 2 and further endorsed by the WHO 3. Reviewing of 4,790 PTCs from Canada, Parente et al. 8 identified 102 cases using strict criteria for NIFTP (2.1% of all PTCs). Although the overall clinical behavior was favorable, 5 of the 102 patients (4.9%) presented lymph node metastasis and one case had a distant metastasis 8.

Kim et al. 9 studied 74 NIFTPs from Korea and identified lymph node metastasis in 9 (12%). However, 5 of their 9 cases had coexisting PTCs 9, and thus this number may not be representative.

Cho et al. 10 assessed the impact of finding a minor percentage of true papillae mixed with the neoplastic follicles in a cohort of 152 EFV-PTCs. Of this group, 105 cases had isolated papillae, but still <1% as defined by criteria for invoking the diagnosis of NIFTP; 3 (3%) patients presented with central lymph node metastasis, 1 (1%) patient had distant metastasis, while at a molecular level, 10 (10%) of the tumors showed *BRAF* V600E mutation. If the stricter criterion of *no papillae* was applied, all cases with *BRAF* V600E mutation were eliminated, leaving 95 cases without invasion, but still 2 (3%) patients had central lymph node metastasis 10.

The authors of this editorial, written on behalf of the members of the NIFTP working group 2, wish to reiterate that the histological criteria for NIFTP must be strictly adhered to in order to keep this category as pure as possible, undiluted by more classical papillary carcinomas that show a characteristic fully malignant potential. Specific consideration must be given to the following criteria:

- **Presence of papillae**: Although the original concept was that some isolated hyperplastic-type papillae could be accepted, the criterion of <1% of papillae was used to highlight how limited the papillary structures should be. It appears that this criterion has been misinterpreted and expanded to allow well-formed papillary structures to be present. Thus, we believe that this exclusion criterion should be modified to state that “no true papillary structures” are acceptable for diagnosing NIFTP. When papillae are present, another diagnosis (such as encapsulated noninvasive PTC or classical PTC) would be more appropriate.
- **Fully-developed nuclear features of PTC**: When using the visual guide (Figure 1), a nuclear score of 2 or 3 was required for the diagnosis of NIFTP. However, in widespread clinical application, it is important to realize that most cases of NIFTP show a nuclear score of 2 rather than 3. Thus, if the case has florid nuclear features of papillary carcinoma (i.e., nuclear score of 3), additional effort should be undertaken to be certain there are no papillae or invasion. This should include examination of the entire capsule and also the whole tumor.
- **Sufficient sections submitted**: Review of the cases received in consultation since the introduction of NIFTP has demonstrated many cases without a full representation of the tumor-to-capsule-to-parenchymal interface. Unless more sections to include the entire periphery of the tumor can be submitted, we recommend not to diagnose NIFTP, and instead render a diagnosis of EFVPTC. The entire capsule should be examined to confirm this diagnosis. To exclude the presence of a papillae, the entire lesion must be examined.
- **Molecular characteristics**: Definitive molecular profiles for various papillary and follicular neoplasms are not unique nor well developed at present. Thus, NIFTP shows an overlapping molecular signature with other neoplasms. While research on micro-RNA expression and other molecular findings are ongoing, if molecular studies have been performed, and mutations characteristic of conventional PTC or high-risk cancer are identified, such as *BRAF* V600E, *RET/PTC*, or *TERT* the diagnosis of NIFTP should not be made. The typical molecular profile of NIFTP is of *RAS* and other *RAS*-like mutations, and thus other molecular findings may be used to exclude the diagnosis of NIFTP.

In conclusion, we would like reiterate that when appropriately diagnosed, NIFTP is a borderline entity with indolent clinical behavior. However, the histological criteria for NIFTP should be strictly followed in order to keep this category as pure as possible.

## Figures and Tables

**Figure 1:**
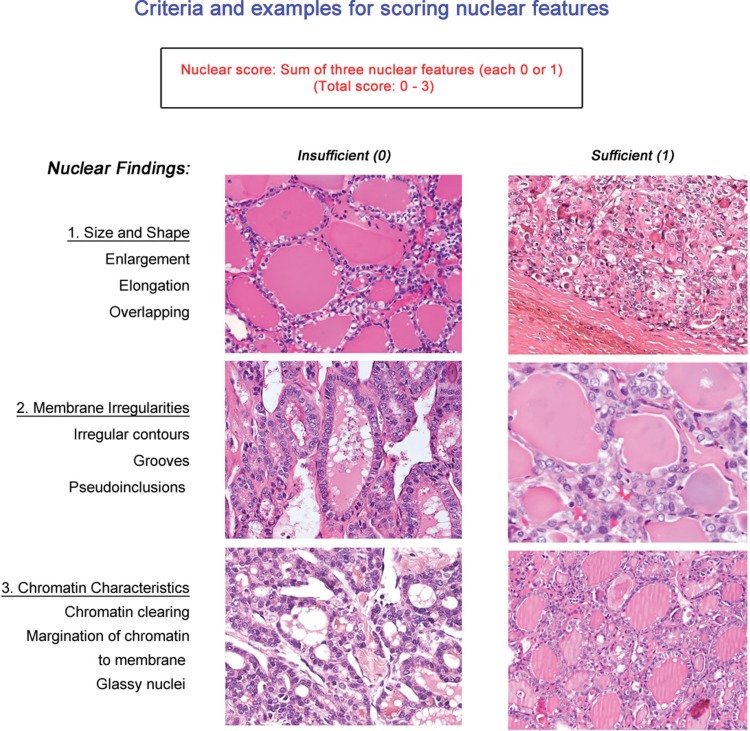
Criteria for scoring nuclear features.
